# DeteX: A highly accurate software for detecting SNV and InDel in single and paired NGS data in cancer research

**DOI:** 10.3389/fgene.2022.1118183

**Published:** 2023-01-06

**Authors:** Yunlong Cui, Hongfeng Li, Pengfei Liu, Hailong Wang, Zhenzhen Zhang, Hongzhu Qu, Caijuan Tian, Xiangdong Fang

**Affiliations:** ^1^ Department of Hepatobiliary Oncology, National Clinical Research Center for Cancer, Key Laboratory of Cancer Prevention and Therapy of Tianjin, Tianjin’s Clinical Research Center for Cancer, Tianjin Medical University Cancer Institute and Hospital, Tianjin, China; ^2^ Department of Clinical Laboratory, Tianjin Academy of Traditional Chinese Medicine Affiliated Hospital, Tianjin, China; ^3^ Department of Oncology, Tianjin Academy of Traditional Chinese Medicine Affiliated Hospital, Tianjin, China; ^4^ Tianjin Marvel Medical Laboratory, Tianjin Marvelbio Technology Co., Ltd., Tianjin, China; ^5^ Beijing Institute of Genomics, Chinese Academy of Sciences/China National Center for Bioinformation, Beijing, China

**Keywords:** snv/InDel caller, NGS, substitution and complex mutations, substitution, mutations

## Abstract

**Background:** Genetic testing is becoming more and more accepted in the auxiliary diagnosis and treatment of tumors. Due to the different performance of the existing bioinformatics software and the different analysis results, the needs of clinical diagnosis and treatment cannot be met. To this end, we combined Bayesian classification model (BC) and fisher exact test (FET), and develop an efficient software DeteX to detect SNV and InDel mutations. It can detect the somatic mutations in tumor-normal paired samples as well as mutations in a single sample.

**Methods:** Combination of Bayesian classification model (BC) and fisher exact test (FET).

**Results:** We detected SNVs and InDels in 11 TCGA glioma samples, 28 clinically targeted capture samples and 2 NCCL-EQA standard samples with DeteX, VarDict, Mutect, VarScan and GatkSNV. The results show that, among the three groups of samples, DeteX has higher sensitivity and precision whether it detects SNVs or InDels than other callers and the F1 value of DeteX is the highest. Especially in the detection of substitution and complex mutations, only DeteX can accurately detect these two kinds of mutations. In terms of single-sample mutation detection, DeteX is much more sensitive than the HaplotypeCaller program in Gatk. In addition, although DeteX has higher mutation detection capabilities, its running time is only .609 of VarDict, which is .704 and .343 longer than VarScan and MuTect, respectively.

**Conclusion:** In this study, we developed DeteX to detect SNV and InDel mutations in single and paired samples. DeteX has high sensitivity and precision especially in the detection of substitution and complex mutations. In summary, DeteX from NGS data is a good SNV and InDel caller.

## Graphical Abstract

**Figure:**
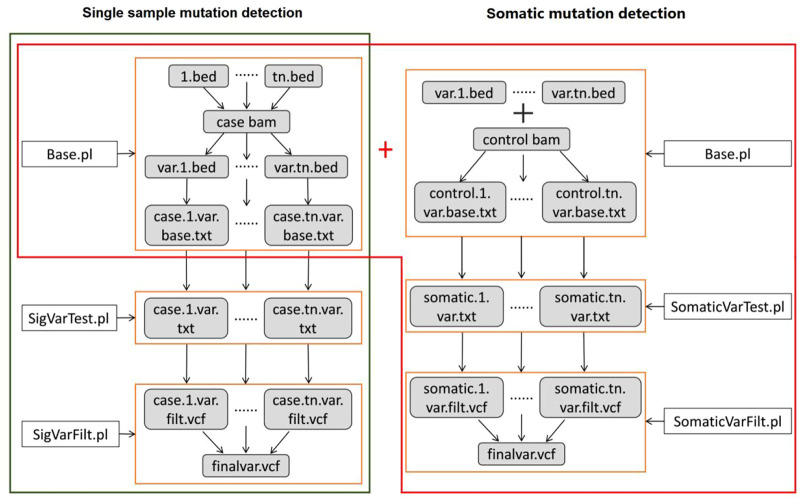
Software running process and output results.

## Background

After a long period of extensive and intensive research, it was believed that tumor is a disease driven by genetic mutations, which is closely related to clinical diagnosis and personalized therapy (Stratton, 2011; Ding et al., 2012; Frampton et al., 2013; Kandoth et al., 2013; Landau et al., 2013). Although the next-generation sequencing (NGS) has become more widely accessible in cancer research for its high-throughput advantages, the accuracy of this technology depends to a large extent on the optimization of data analysis. Cancer genome analysis is expected to reveal the patterns of genetic alterations, including single nucleotide variantions (SNVs), multi-nucleotide variantions (MNVs), insertion and deletions (InDels), complex variantions etc. Among all mutation types, SNVs and InDels get the most attention from variant callers (McKenna et al., 2010; Koboldt et al., 2012; Cibulskis et al., 2013; Lai et al., 2016). Due to the variable sample material, the rare frequency of alteration and the complex mutation event, it is a tough and urgent need to accurately detect mutations from NGS data (Stransky et al., 2011; Banerji et al., 2012; Carter et al., 2012).

Errors in bioinformatics processes and experimental steps may confuse the real variants of clinical samples (Robasky et al., 2014; O'Rawe et al., 2013; Kircher et al., 2011; Metzker, 2010). As a consequence, several regularly cited tools such as VarScan2, VarDict, MuTect and GATK, have been developed to solve or partially solve these problems. VarScan2 and VarDict apply the Fisher’s Exact Test (FET) to detect mutations (Koboldt et al., 2012; Lai et al., 2016). Even though FET performs well at calling crucial and slight variables that other callers are likely to miss or ignore, the accuracy is still insufficient due to inadequate false positive filtering. In addition, VarDict’s versatile capability drags down its running speed performance at computing the specific type of mutation.

Another statistical method is using Bayesian Classifier (BC) to detect somatic point alterations (Cibulskis et al., 2013), such as MuTect. MuTect is a highly sensitive mutation caller, only requiring a few supporting reads to detect, and a series of filters are used to ensure its specificity. Meanwhile, MuTect applies severe penalties to somatic variant candidates if the variant sites are also found in the matched normal. While this approach filters out the most false positives of germline variants, it adversely affects sensitivity in cases when the normal sample is contaminated. GATK is a comprehensive variants caller that combines multiple methods to detect germline or somatic mutations (McKenna et al., 2010). However, GATK has poor sensitivity and accuracy in detecting low-frequency variants, especially those with variant allele frequencies (VAFs) less than 5%. Generally, most of these approaches would be confounded by false negative or false positives owing to impure sample compositions, deviations in experimental operation and imperfect detecting strategies.

To address these problems mentioned above, we have developed a high-confidence variant caller, DeteX which can detect variants in tumor-only or tumor-normal matched NGS data. This software is developed by integrating FET and BC algorithms in Perl language to adapt to different conditions and purposes. Considering the artifacts caused by polymerase chain reaction (PCR) and mutations occurring around InDels, many filtering conditions were optimized to ensure the accuracy of detecting mutations, and the efficiency of DeteX is improved by running sub-regions in parallel. In this study, *F*1 score was used as a quantitative indicator of accuracy to compare the performance of DeteX with that of other callers. Moreover, three group sample datasets, including 11 pairs of TCGA glioma samples, 28 pairs of real tumor targeted sequencing samples and two standard samples from External Quality Assessment of High-throughput Sequencing for Tumor Somatic Mutation in China (NCCL-EQA), were used to assess the performance of DeteX. This study proved that DeteX can improve the accuracy of mutation detection, especially in detecting substitution and complex mutations, and it could be used as a convenient tool to replace the multiplex calling and filtration pipeline.

## Materials and Method

### Datasets

In order to evaluate the performance of DeteX, we used a range of real and standard datasets as shown below:

1 The public whole genome sequencing datasets from TCGA: 11 pairs of glioma data in sam format, with average sequencing depth of 50X-100X. The variant set of Mutect2 software for these 11 samples (Supplementary Table S2) was obtained from the TCGA (https://portal.gdc.cancer.gov/) website was used as the standard variant set for subsequent analysis.
2 The real clinical targeted sequencing data: Blood from 28 pairs from clinical patients with lung cancer and intestinal cancer were selected. ctDNA from blood samples was used as tumor samples, and leukocytes were used as normal samples. These DNA samples were extracted, library was constructed and sequenced to obtain sequencing data in fastq format.

The data were obtained by the process of filtering, Bwa (.7.12-r1039) (McKenna et al., 2010) comparison, marker duplication by Picard MarkDuplicates.jar (1.119) package, re-matching by Gatk (Lai et al., 2016) and correction of base mass values to obtain the bam data of the samples. The average sequencing depth of samples ranged from 500X to 2500X.

3 The NCCL-EQA data: Illumina Hiseq platform data of NCCL-EQA in 2017 and 2019 were analyzed to obtain bam data through the same process of reads filtering, alignment, marking duplication, realignment and correction of base quality value.

### Requirements and implementation

DeteX starts with Binary Alignment/Map (BAM) file, which is generated from NGS reads alignment or procedures, such as BWA (Li and Durbin, 2009), TopHat (Trapnell et al., 2009; Eenst et al., 2017), Bowtie (Langmead et al., 2009), and Bowtie2 (Langmead and Salzberg, 2012; Langmead et al., 2019). It is developed in Perl language. The source code can be downloaded at https://github.com/mvlzwtd/DeteX. This website is maintained by Marvelbio O&M team.

### Structure and workflow

**FIGURE 1:**
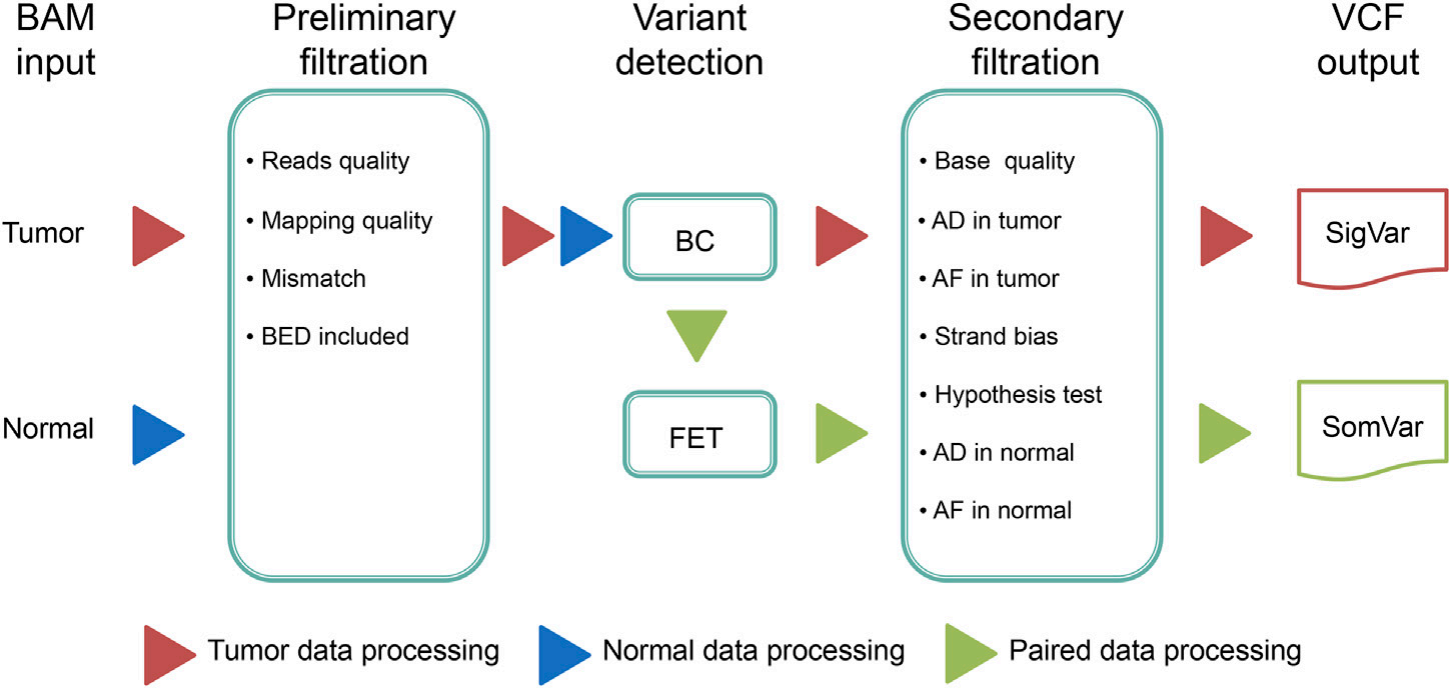
The workflow of DeteX. DeteX uses BAM format file as input. The preliminary filtering is mainly concerned with base quality and mapping quality. Then it applies Bayesian classifier (BC) method for mutation calling. For tumor-normal matched data, Fisher’s exact test (FET) is further performed to identify the candidate variant. By reducing false positive by meeting several screening criteria (Supplementary Table S1) the final high-confidence variants is obtained. SigVar indicates single tumor variant and SomVar indicates somatic variant.

DeteX can detect somatic and germline mutations in tumor-only or somatic mutations in tumor-normal paired samples. The detection strategy includes the following steps:

(i) Filtering reads with low base quality, low mapping quality, duplicates, multiple mapped and outside of the detection interval.
(ii) Detecting variants, if there is a control sample, the process of filtering variation should be added to the control sample.
(iii) After filtering the false positive variants based on multiple filtering conditions, high confidence variants are ultimately output. The workflow of DeteX is illustrated in Figure 1.

## Variant detection principle

### Reads filtering

In order to reduce the number of false positive variants caused by non-independence errors and sequencing errors, we have set various filtering conditions in our software, which are derived from the experience of developers in data analysis and suggestions of other software. The filtering conditions applied in our software, including reads and variants filtering, are shown in Supplementary Table S1. These conditional values can be set by parameters except for mapping type.

Meeting all the reads filtering and the first 4 variants filtering conditions in Supplementary Table S1 and then these reads can be used to detect mutations by the following BC and FET model.

### SNV detection

We calculate the ratio of the maximum likelihood values (LOD) of the two models for each variant locus by using the model introduced in the MuTect supplemental method (Cibulskis et al., 2013). The larger LOD value of a variant, the more reliable it is. We calculate the LOD value for each variant in the tumor sample, and filter out the variants with LOD value less than 3.9.

If there is no control sample, perform variations filtering according to the conditions 5, 6, and 7 in Supplementary Table S1, and output the final variations in VCF format. The results contain germline and somatic variants. If there is no control samples, all we need is the results of somatic mutation. We can screen out somatic mutations by dbSNP (Smigielski et al., 2000; Sherry et al., 2001; Chiang et al., 2017; Arifuzzaman et al., 2020) (https://www.ncbi.nlm.nih.gov/snp/), Cosmic (Forbes et al., 2017) (https://cancer.sanger.ac.uk/cosmic), 1000G (https://www.ncbi.nlm.nih.gov/variation/tools/1000genomes/), gnomAD (http://hgdownload.cse.ucsc.edu/gbdb/hg19/gnomAD/vcf/), EXAC (Song et al., 2016) (http://exac. broadinstitute.org) or other databases. The dbSNP, 1000G, gnomAD and EXAC databases are healthy human variant databases. If this variant exists in these databases, and the population variant frequency is greater than or equal to 1%, then this variant is considered as a germline variant, otherwise it is considered as somatic mutation. Cosmic database is database of tumor variants. If there is a variant, it is considered to be somatic mutation. If a variant is considered as both somatic and germline variant, it means that both possibilities exist.

If there is a control sample, the frequency of variation in the control sample needs to be less than the value of parameter “-nf” or .2 times frequency in the tumor sample. The variants that are not in control sample or supporting reads in the same direction are directly output. The LOD value of a variation in the control sample whose frequency is less than .5 times that in the tumor is calculated. If the LOD value is less than 3.9, the variation will be output. If it is greater than or equal to 3.9, the significance *p*-value of the variation is calculated according to Fisher’s exact test. If the *p*-value is less than or equal to the parameter setting value, the variation is output, otherwise it is not output. The *p*-value of Fisher’s exact test of a variation is calculated as follows: We denote supporting variant reads in normal sample as $n_{1}$ , supporting reference reads in normal sample as $n_{0}$ , supporting variant reads in tumor sample as $c_{1}$ , supporting reference reads in normal sample as $c_{0}$ . The significance value *p* is given by

$$p=\frac{\left(n_{1}+n_{0}\right)!\left(c_{1}+c_{0}\right)!\left(c_{1}+n_{1}\right)!\left(c_{0}+n_{0}\right)!}{n_{1}!n_{0}!c_{1}!c_{0}!\left(n_{1}+n_{0}+c_{1}+c_{0}\right)!}$$

Next, according to the conditions 5, 6, 7. and 8 in Supplementary Table S1, variation filtering is performed, and then the final variations in VCF format are outputted. If two adjacent loci are mutated at the same time, which shows that they are mutated in the same reads, we call this type mutation a substitution mutation. According to this features, we can correctly output these type mutations. Delete reads with more than or equal to three mutations, most of which are caused by alignment errors.

### InDel detection

The software applies Fisher’s exact test algorithm to detect InDel. After meeting the reads filtering conditions, reads with one or two gaps/insert sequences (inss) are retained. If SNV variants exist SNV in the same reads within five bases distance to gap/ins, they are combined into one variant. If the gap or the distance among the gaps or inss in the same reads is less than 10 bases, they are also combined into a variant. If the two mutations are in the different reads, they are considered to be two variants. These conditions ensure the detection of complex mutation correctly. Variant filter conditions 5, 6, 7, 8, 9, and 10 in Supplementary Table S1 are also applicable to InDel detection.

### Variation frequency calculation

Due to PCR amplification, duplicate reads may occur at the variant site. Duplicate reads are counted as one in variation frequency calculation. In repeated reads, if the proportion of mutated reads is greater than .8, it is considered as a read supporting variation. The maximum base quality value of mutated reads at the variation site is considered as the base quality value of this site. Only reads that cover all the InDel sites are counted in InDel frequency calculation. Final variation frequency VAF is given by

$$VAF=\frac{total\;mutation\;reads}{total\;reads}\times 100\%$$

### Softwares to detect SNV/InDel

To evaluate the performance of DeteX, we used DeteX, VarScan, MuTect and VarDict to detect SNV and DeteX, VarScan, Gatk and VarDict to detect InDel. In the detection of SNVs and InDels of three groups of datasets, DeteX and Mutect were used the default value of the parameters. We added parameters of “--min-avg-qual 13, --min-tumor-freq .01, --max-normal-freq .02” to VarScan, “-filter T_INDEL_F < .01” to Gatk, “-m 5 -O 40 -V .02 -x 0 -k 0 -X 3 -c 1 -S 2 -E 3 -g 4” to VarDict.

### Performance evaluation metrics

We use sensitivity, precision and F1 value to evaluate the performance of the software. Sensitivity is the proportion of detected true variantions in all the true positive variations. The higher the sensitivity, the lower the rate of missed detection. Precision is the proportion of detected true variantions in all the observed variations. The higher the precision is, the accuracy is higher. The value of F1 is the products of sensitivity and precision, which comprehensively reflects the performance of variation detection of softwares.

## Results

### Benchmark variant datasets

Benchmark variant data is very important for assessing the performance of variant detection software. A few typical real samples containing all types of mutations, and simulation data usually can not contain all kinds of random errors in sequencing, public authority samples and standard samples can be used to evaluate software performance. In this paper, three groups of samples are selected. Each group of sample has different ways to get the benchmark variant data.

11 TCGA samples. The Mutect2 software can obtain the most reliable variant results. So we used variants detected by Mutect2 as the final benchmark variant dataset. The mutations with sequencing depth below eight and less than 2 variant reads are dropped. Finally, 760 mutations, including 37 InDels and 723 SNVs (Supplementary Table S2) were found in 11 glioma samples with Mutect2-labeled. Because substitution and complex mutations are considered as two mutations in the public mutation database, 763 variants are displayed in the table. We put the substitution mutations into the SNV set and the complex mutations into the InDel set.

28 real clinical samples. All SNVs and InDels with variant frequency ≥1% were detected. Those detected by at least two softwares were put into the final benchmark variant set. The categorization method is the same as TCGA sample variants, with substitution mutations categorized as SNV and complex mutations categorized as InDel. There are 1,481 variants (SSupplementary Table S3) including 1,045 SNVs and 436 InDel in the benchmark variant set.

NCCL-EQA external quality assessment data. There is a set of standard variables to assess the testing capabilities of laboratories across China. 94 variants are available in 2017 sample (Supplementary Table S4) and 23 variants are available in 2019 sample (Supplementary Table S5). These standard datasets enable accurately evaluate the performance of the software.

### Higher precision and sensitivity of DeteX in SNVs and InDel detection

We compared the accuracy and sensitivity of each software in detecting SNVs and InDels in 11 pairs of TCGA samples (Table 1). In the table, in the SNV detection, VarScan.hc and MuTect have the best precision but at the expense of sensitivity. In contrast, VarScan has the best performance in sensitivity, but it contains a large number of false positive variants, resulting in the lowest accuracy. VarDict and DeteX show relatively balanced in both metrics, but DeteX slightly outperforms vardict in all metrics. So the score is the highest among all the software when it measured by F1 values. In the InDel detection, VarScan has a similar performance in detecting SNV with the lowest accuracy. VarScan.hc has a relatively high precision but has the lowest sensitivity. DeteX has great advantage over other software in all metrics, and it can reach a maximum F1 of one.

**TABLE 1:** Results of each caller to detect variants in TCGA samples.

Mutation type	Caller	Benchmark mutation num	Detected mutation num	True positive mutation num	Precision (%)	Sensitivity (%)	F1
SNV	VarDict	723	681	673	98.83	93.08	.96
	DeteX		686	684	99.71	94.61	.97
	VarScan.hc		610	610	100.00	84.37	.92
	VarScan		934	707	75.70	97.79	.85
	MuTect		654	654	100.00	90.46	.95
InDel	VarDict	37	39	35	89.74	94.59	.92
	DeteX		37	37	100.00	100.00	1.00
	VarScan.hc		31	29	93.55	78.38	.85
	VarScan		49	35	71.43	94.59	.81
	Gatk		44	35	79.55	94.59	.86

**FIGURE 2:**
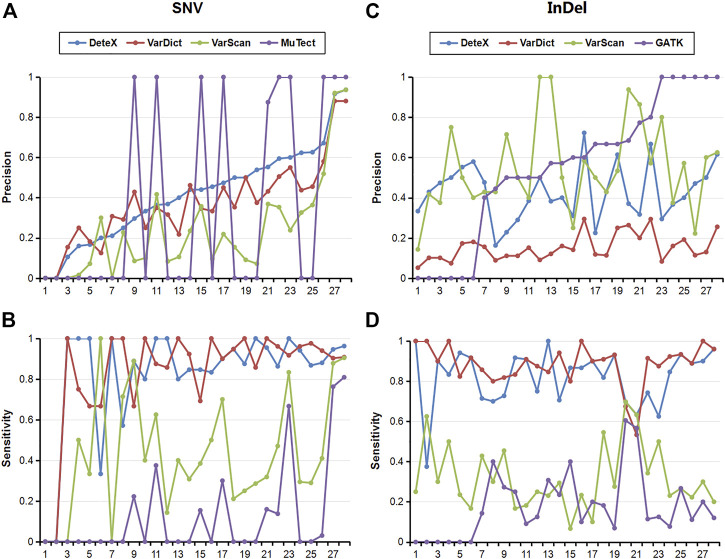
Sensitivity and precision of each software for the detection of SNV and InDel in 28 clinical samples. **(A)** Precision of SNVs, **(B)** Sensitivity of SNVs, **(C)** Precision of InDels, **(D)** Sensitivity of InDels.

We also compared the results of each software for SNVs and InDels in 28 pairs of clinically targeted sequencing samples (Table 2; Figure 2). As shown in Table 2, DeteX and VarDict have similar sensitivity in SNV detection and they are significantly higher than VarScan and MuTect. The accuracy of DeteX is lower than MuTect, but higher than VarDict and VarScan.Finally DeteX has the highest F1 value. In the InDel detection, DeteX and VarDict have similar sensitivity, which is significantly higher than VarScan and Gatk. The accuracy of DeteX is moderate. The final the F1 value of DeteX is also the highest. Figure 2 shows the sensitivity and accuracy of each software in detecting SNV and InDel in 28 samples in more intuitive and detailed way. In some samples, there is no variant detected by MuTect. The precision of it in these samples is marked 0, so the status of 1 and 0 are appeared in Figure 2A. All variants marked somatic including “StrongSomatic” and “LikelySomatic” are counted in the VarDict results. Therefore, in InDel detection, the precision of VarDict performs the worst (Figure 2C). DeteX and VarDict are significantly better than the other two softwares in terms of sensitivity both in detecting SNV and InDel (Figures 2B, D).

**TABLE 2:** Results of each caller to detect variants in clinical samples.

Mutation type	Caller	Detected mutation num	True positive mutation num	Benchmark mutation num	Precision	Sensitivity	F1
SNV	DeteX	1,445	968	1,045	.67	.93	.62
	VarDict	1,637	962		.59	.92	.54
	VarScan	1,424	693		.49	.66	.32
	Mutect	485	484		1.00	.46	.46
InDel	DeteX	854	355	436	.42	.81	.34
	VarDict	2371	375		.16	.86	.14
	VarScan	260	151		.58	.35	.20
	Gatk	152	96		.63	.22	.14

We also analyzed the variant results of each software for the NCCL-EQA external quality assessment samples. Each software could detect SNVs accurately (Supplementary Table S6). For InDel, substitution and complex mutations (Table 4), only DeteX detected all of them correctly, VarDict detected 22 and both VarScan and Gatk detected 16.

### DeteX detects substitutions and complex mutations more accurately

**FIGURE 3:**
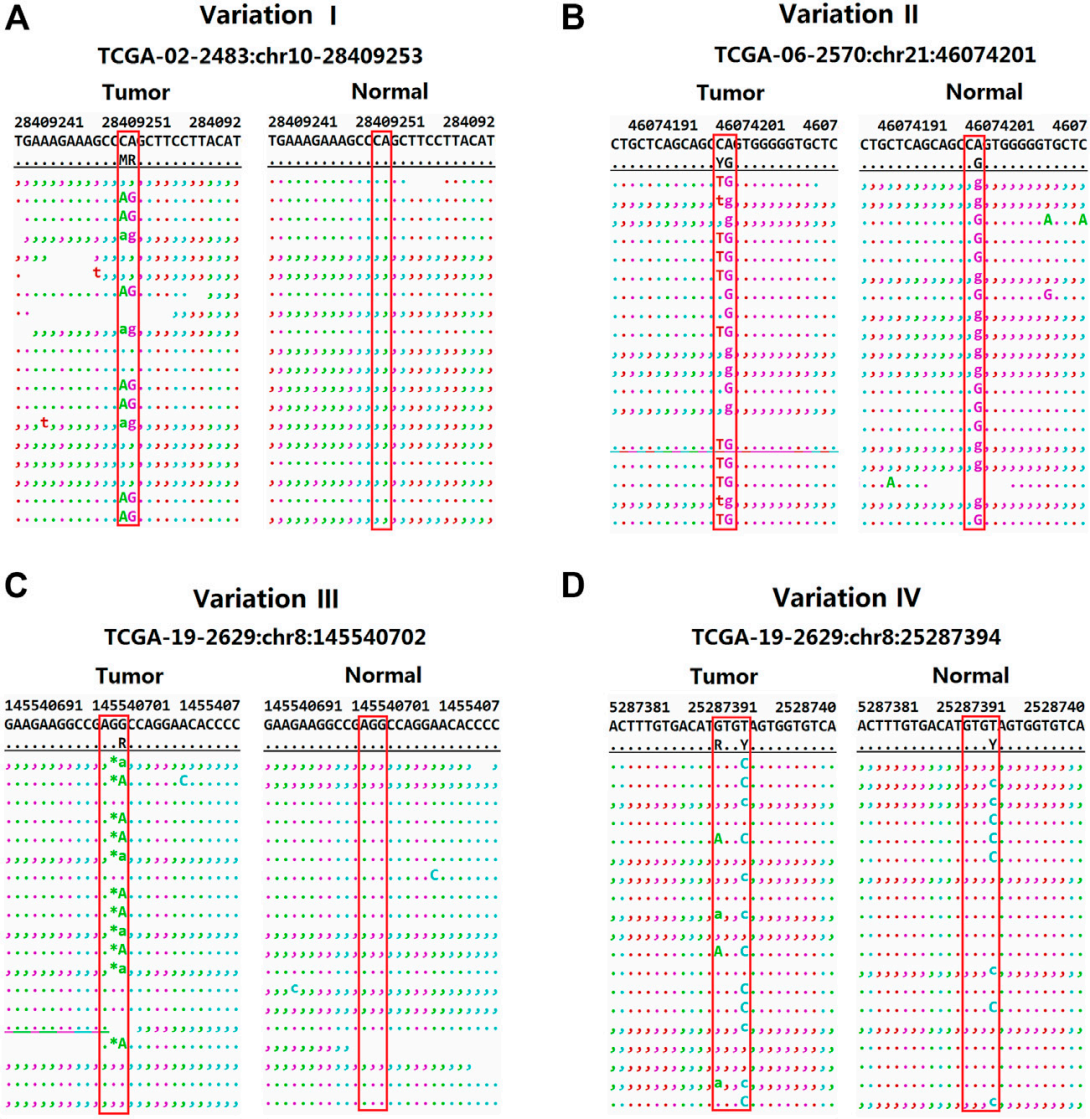
The distribution of reads for four variants in the TCGA sample (obtained by samtools tview program). **(A)** The distribution of reads for CA->AG in the TCGA sample. **(B)** The distribution of reads for C->T in the TCGA sample. **(C)** The distribution of reads for GC->A in the TCGA sample. **(D)** The distribution of reads for G->A in the TCGA sample.

It shows clearly in Table 3 and Figure 3 to detect substitution and complex mutations in TCGA samples by each software. From these results, it can be seen that for substitution mutations such as the variant one in Table 3 (Figure 3A), two adjacent bases are mutated in the same reads, and the standard result is chr10:28409253–28409254, CA- > AG, which was detected as two adjacent mutations by VarScan and MuTect, but accurately detected by VarDict and DeteX. If two adjacent bases are mutated, but one of them also mutated in the normal sample, it is not a substitution mutation. For example, the standard result of the variant 2 in Table3 (Figure 3B) is chr21:46074201, C- > T. VarDict detected as CA- > TG, the other softwares detected correctly. The above two cases indicate that VarScan, MuTect and VarDict have certain detection errors for the mutations occurring in adjacent sites. For complex mutations, variant three in Table3 (Figure 3C) is a deletion accompanied by a SNV variant. The standard result is chr8:145540703, GG>A.The results show that only DeteX could detect it correctly. VarDict detected it as two mutations. VarScan and Gatk detected a deletion only. MuTect did not detect the mutation. The variant 4 in Table 3 (Figure 3D) is similar to the variant 2 except that the two mutanted loci are not adjacent to each other. So the result is the same as the variant 2.

**TABLE 3:** Four substitution and complex variations in TCGA glioma data.

Caller	Variation I				Variation II				Variation III				Variation IV			
	Chr	Pos	Ref	Var	Chr	Pos	Ref	Var	Chr	Pos	Ref	Var	Chr	Pos	Ref	Var
VarScan	chr10	28409253	C	A	chr21	46074201	C	T	chr8	145540703	G	—	chr8	25287394	G	A
	chr10	28409254	A	G												
Mutect	chr10	28409253	C	A	chr21	46074201	C	T	—	—	—	—	chr8	25287394	G	A
	chr10	28409254	A	G												
VarDict	chr10	28409253	CA	AG	chr21	46074201	CA	TG	chr8	145540703	G	—	chr8	25287394	GTGT	ATGC
									chr8	145540704	G	A				
Gatk	—	—	—	—	—	—	—	—	chr8	145540703	G	—	—	—	—	—
DeteX	chr10	28409253	CA	AG	chr21	46074201	C	T	chr8	145540703	GG	A	chr8	25287394	G	A

**FIGURE 4:**
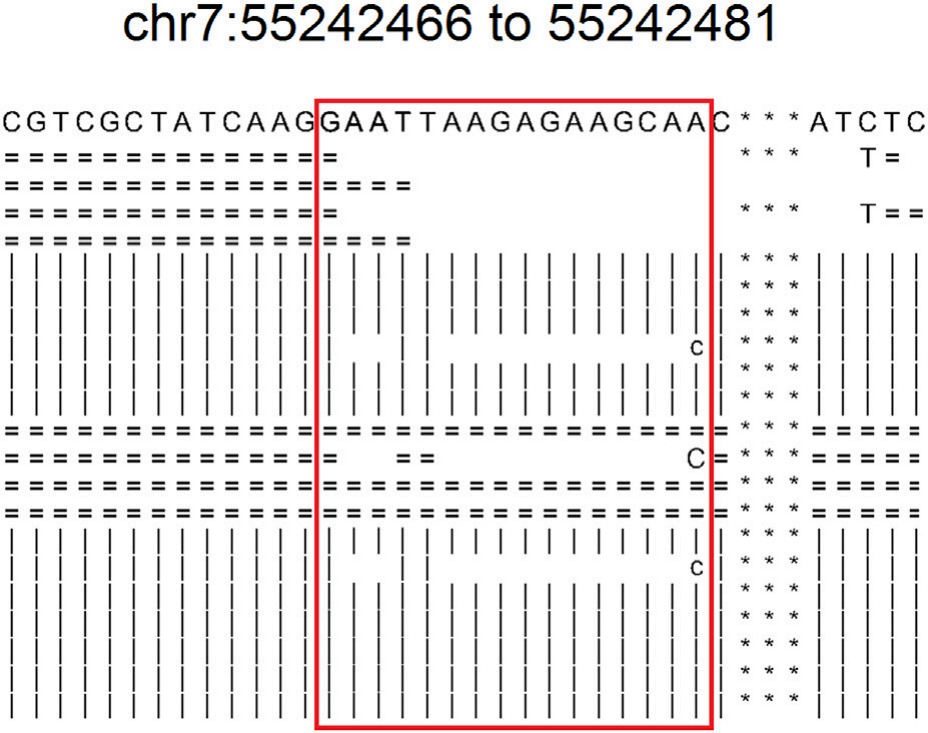
The readsmapping of one complexmutation. The sites in the red box represents from 55242466 to 55242481 of chr7. “ = ” indicates that it is identical to the reference base in the positive strand. “|” indicates that it is identical to the reference base in the negative strand. “c” indicates negative strandmismatch. “C” indicates positive strand mismatch. Blank indicates deletion.

The results of NCCL-EQA (Table 4) once again confirmed the excellent detection ability of DeteX for complex mutations and substitution mutations. Only DeteX detected all of them. VarDict could accurately detect substitution mutations, while complex mutations can only be partially detected or not. The other two software could partially detect or fail to detect these two kinds of mutations. The detection ability of DeteX is excellent especially in complex mutations which contain SNV and gaps. For example, four events AAdel, TTmap, AAGAGAAGCAdel, and A- > C occurred simultaneously in the mutation chr7: 55242467–55242481 AATTAAGAGAAGCAA- > TTC (Figure 4). It is difficult to output it accurately without considering the adjacent InDel and SNV together.

**TABLE 4:** The results of InDel, substitution, and complex mutations in two NCCL-EQA samples by each software.

SampleID	Num	Chr	Start	End	Ref	Var	Gene	Type	VAF (%)	VarScan	GATK	DeteX	VarDict
201711	1	1	115256520	—	—	CCCGGCAC	NRAS	Insertion	2	√	√	√	√
	2	2	29445271	—	—	CGT	ALK	Insertion	15	√	√	√	√
	3	3	37089070	37089072	ACA	—	MLH1	Deletion	5	√	√	√	√
	4	3	41266107	41266108	TC	AA	CTNNB1	Complex	2	×	×	√	√
	5	3	178916946	178916948	GAT	—	PIK3CA	Deletion	7	√	—	√	√
	6	4	55152092	55152100	GACATCATG	—	PDGFRA	Deletion	6	—	√	√	√
	7	4	106180857	106180857	C	—	TET2	Deletion	10	√	√	√	√
	8	5	112175210	—	—	A	APC	Insertion	6	√	√	√	√
	9	7	55242467	55242481	AATTAAGAGAAGCAA	TTC	EGFR	Complex	3	×	—	√	×
	10	7	140453132	140453136	TTTCA	AT	BRAF	Complex	5	—	×	√	×
	11	9	5070022	5070027	TCACAA	—	JAK2	Deletion	4	√	√	√	√
	12	10	89692837	89692842	TCTTGA	—	PTEN	Deletion	2	√	-	√	√
	13	10	123247618	123247620	GAT	—	FGFR2	Deletion	3	√	√	√	√
	14	11	32417910	—	—	ACCGT	WT1	Insertion	5	√	√	√	√
	15	11	64575435	—	—	CTGT	MEN1	Insertion	5	√	√	√	√
	16	11	108170483	108170487	TCTCT	—	ATM	Deletion	8	√	√	√	√
	17	12	25380259	—	—	TGCACTGTACTCCTC	KRAS	Insertion	3	√	√	√	√
	18	13	28608104	—	—	AAGCACCTGATCCTAG TACCT	FLT3	Insertion	7	√	√	√	√
	19	17	7577105	—	—	GA	TP53	Insertion	13	√	√	√	√
	20	22	30032780	30032801	GGA​CTC​TGG​GGC​TCC​GAG​AAA​C	—	NF2	Deletion	3	—	—	√	√
201911	1	2	148683693	148683693	A	—	ACVR2A	Deletion	16	√	√	√	√
	2	2	209113112	209113113	CG	GA	IDH1	Complex	14	×	×	√	√
	3	7	55242470	55242495	TAA​GAG​AAG​CAA​CAT​CTC​CGA​AAG​CC	CGAAAGG	EGFR	Complex	15	×	×	√	×
	4	7	140453135	140453136	CA	GT	BRAF	Complex	24	×	×	√	√
	5	20	31022449	—	—	G	ASXL1	Insertion	27	—	√	√	√

√’ indicates a correct result, “-” indicates no result, and “×” indicates part of result.

### Single sample testing

We analyzed the SNVs and InDels results of DeteX and the HaplotypeCaller (DePristo et al., 2011) program in Gatk in tumor sample from the 2017 NCCL-EQA external quality assessment (Table 5). DeteX detected all the 94 variants in the standard variant set, but only 29 variation were detected by HaplotypeCaller, most of which were more than 10%. This indicates that DeteX is more sensitive than HaplotypeCaller in detecting somatic variants in a single sample.

**TABLE 5:** The results of DeteX and HaplotypeCaller to detect variants in tumor sample from the 2017 NCCL-EQA.

Chr	Start	End	Ref	Obs	Gene_Symbol	MType	Freq (%)	Gatk	DeteX
chr1	10412784	10412785	A	G	KIF1B	SNV	3.08	—	√
chr1	11190665	11190666	C	T	MTOR	SNV	5.94	—	√
chr1	43812575	43812576	T	C	MPL	SNV	6.8	—	√
chr1	115256520	—	—	AGGCCAGG CCCGGCAC TG	NRAS	Insertion	1.7	—	√
chr2	29445271	—	—	CGT	ALK	Insertion	13.72	√	√
chr2	29519890	29519891	C	G	ALK	SNV	6.16	—	√
chr2	29541234	29541235	C	T	ALK	SNV	6.12	—	√
chr2	209108165	209108166	T	C	IDH1	SNV	3.29	—	√
chr2	212566836	212566837	C	A	ERBB4	SNV	3.48	—	√
chr3	37089070	37089072	ACA	—	MLH1	Deletion	4.38	—	√
chr3	41266107	41266108	TC	AA	CTNNB1	Complex	1.82	—	√
chr3	47164278	47164279	G	A	SETD2	SNV	7.49	√	√
chr3	142224005	142224006	C	A	ATR	SNV	9.17	√	√
chr3	142232475	142232476	C	T	ATR	SNV	3.99	—	√
chr3	178916946	178916948	GAT	—	PIK3CA	Deletion	7.59	√	√
chr3	187447267	187447268	C	T	BCL6	SNV	14.39	√	√
chr4	1805528	1805529	T	G	FGFR3	SNV	5.01	—	√
chr4	1808929	1808930	G	A	FGFR3	SNV	3.79	—	√
chr4	55131212	55131213	A	T	PDGFRA	SNV	17.99	√	√
chr4	55152092	55152100	GACATCATG	-	PDGFRA	Deletion	5.61	—	√
chr4	68610385	68610386	G	T	GNRHR	SNV	13	√	√
chr4	106180857	106180857	C	-	TET2	Deletion	9.39	√	√
chr4	106190823	106190824	T	C	TET2	SNV	11.83	√	√
chr4	106197175	106197176	G	A	TET2	SNV	11.3	√	√
chr5	112175210	—	—	A	APC	Insertion	5.7	—	√
chr5	112176361	112176362	C	T	APC	SNV	5.23	—	√
chr5	149501448	149501449	G	A	PDGFRB	SNV	4.31	—	√
chr5	176517792	176517793	G	A	FGFR4	SNV	6.2	—	√
chr5	180057074	180057075	C	T	FLT4	SNV	13.05	√	√
chr6	30862397	30862398	G	A	DDR1	SNV	2.17	—	√
chr6	117700257	117700258	C	T	ROS1	SNV	5.66	—	√
chr6	117714444	117714445	C	T	ROS1	SNV	11.47	√	√
chr7	55242467	55242481	AATTAAGAGAAGCAA	TTC	EGFR	Complex	1.2	—	√
chr7	55249037	55249038	G	A	EGFR	SNV	2.46	—	√
chr7	55249091	55249092	G	C	EGFR	SNV	3.53	—	√
chr7	55259514	55259515	T	G	EGFR	SNV	2.16	—	√
chr7	116414979	116414980	A	T	MET	SNV	7.79	—	√
chr7	140453132	140453136	TTTCA	AT	BRAF	Complex	4.93	—	√
chr7	140494237	140494238	G	A	BRAF	SNV	1.47	—	√
chr8	92982976	92982977	C	T	RUNX1T1	SNV	21.35	√	√
chr8	128753093	128753094	C	A	MYC	SNV	4.48	—	√
chr9	5070022	5070027	TCACAA	-	JAK2	Deletion	4.07	—	√
chr9	8500789	8500790	C	T	PTPRD	SNV	4.07	—	√
chr9	21970965	21970966	C	A	CDKN2A	SNV	9.2	√	√
chr9	133760366	133760367	C	A	ABL1	SNV	3.07	—	√
chr9	133760951	133760952	A	G	ABL1	SNV	2.16	—	√
chr9	139391525	139391526	G	A	NOTCH1	SNV	6.15	—	√
chr9	139396741	139396742	T	C	NOTCH1	SNV	10.74	√	√
chr9	139409810	139409811	G	T	NOTCH1	SNV	8.93	—	√
chr10	43596167	43596168	G	A	RET	SNV	4.26	—	√
chr10	63851815	63851816	C	T	ARID5B	SNV	3.65	—	√
chr10	89692837	89692842	TCTTGA	—	PTEN	Deletion	2.46	—	√
chr10	89717612	89717613	C	A	PTEN	SNV	2.61	—	√
chr10	123247618	123247620	GAT	—	FGFR2	Deletion	2.67	—	√
chr11	32417910	—	—	ACCGT	WT1	Insertion	5.26	—	√
chr11	32439125	32439126	T	G	WT1	SNV	5.93	—	√
chr11	64573739	64573740	A	G	MEN1	SNV	4.52	—	√
chr11	64575435	—	—	CTGT	MEN1	Insertion	3.63	—	√
chr11	108114802	108114803	C	G	ATM	SNV	5.82	—	√
chr11	108170483	108170487	TCTCT	—	ATM	Deletion	7.41	—	√
chr11	108206581	108206582	A	T	ATM	SNV	2.45	—	√
chr12	6704522	6704523	G	A	CHD4	SNV	1.47	—	√
chr12	25378590	25378591	C	T	KRAS	SNV	11.68	√	√
chr12	25380259	—	—	TGCACTGTACTCCTC	KRAS	Insertion	2.53	—	√
chr12	58144504	58144505	G	A	CDK4	SNV	10.31	√	√
chr12	115109751	115109752	T	A	TBX3	SNV	9.39	√	√
chr13	28599039	28599040	C	T	FLT3	SNV	9.13	√	√
chr13	28608104	—	—	AAGCACCTGATCCTAG TACCT	FLT3	Insertion	4.49	√	√
chr13	28895609	28895610	C	T	FLT1	SNV	7.31	—	√
chr13	28897044	28897045	C	T	FLT1	SNV	7.75	—	√
chr13	48934220	48934221	T	C	RB1	SNV	10.32	√	√
chr13	48937054	48937055	G	A	RB1	SNV	12.29	√	√
chr14	75514889	75514890	G	T	MLH3	SNV	18.81	√	√
chr14	105246489	105246490	C	T	AKT1	SNV	2.07	—	√
chr15	67358628	67358629	G	A	SMAD3	SNV	16.51	√	√
chr15	67457294	67457295	G	T	SMAD3	SNV	17.73	√	√
chr16	2126135	2126136	C	T	TSC2	SNV	5.86	—	√
chr16	3789660	3789661	C	T	CREBBP	SNV	1.33	—	√
chr16	3828109	3828110	C	T	CREBBP	SNV	4.95	—	√
chr17	7574001	7574002	C	T	TP53	SNV	2.84	—	√
chr17	7577105	—	—	GA	TP53	Insertion	11.64	√	√
chr17	29562778	29562779	T	C	NF1	SNV	8.23	—	√
chr17	29576056	29576057	G	A	NF1	SNV	3.05	—	√
chr17	42327859	42327860	C	T	SLC4A1	SNV	4.22	—	√
chr18	48581260	48581261	C	T	SMAD4	SNV	8.08	—	√
chr18	60985848	60985849	C	G	BCL2	SNV	6.8	—	√
chr19	17943615	17943616	T	A	JAK3	SNV	7.35	—	√
chr19	17945516	17945517	T	C	JAK3	SNV	9.5	—	√
chr22	22142671	22142672	G	T	MAPK1	SNV	8.87	√	√
chr22	23523943	23523944	G	A	BCR	SNV	10.45	√	√
chr22	29695597	29695598	G	A	EWSR1	SNV	4.19	—	√
chr22	30032780	30032801	GGA​CTC​TGG​GGC​TCC​GAG​AAA​C	—	NF2	Deletion	1.17	—	√
chr22	41564512	41564513	G	A	EP300	SNV	4.87	—	√
chrX	53440074	53440075	T	G	SMC1A	SNV	15.15	√	√

√’ indicates a correct result and “-” indicates no result.

### Running speed

**FIGURE 5:**
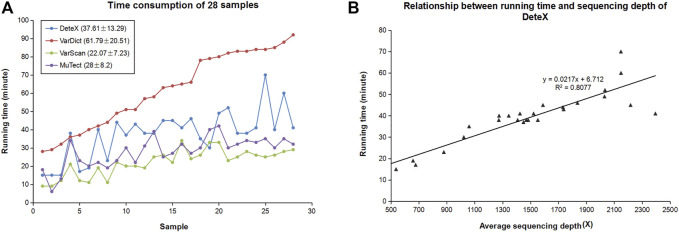
Computational performance of DeteX. **(A)** Time consumption of four callers in 28 clinical samples. **(B)** The relationship between running time of DeteX and average sequencing depth.

Good accuracy is often at the expense of computing time. DeteX increases computing speed by splitting the targeted capture interval by setting the threads. We calculated the time taken by these four softwares to detect SNVs and InDels in 28 pairs of clinical samples (Figure 5A). The samples in Figure 5A are arranged from shortest to longest according to VarDict’s running time. It takes much less time than VarDict. Due to the high complexity of the algorithm, it takes slightly longer than VarScan and MuTect. DeteX processes the bam data linearly first and then it outputs the sequencing reads of the variant site in the case sample. Therefore, the more variations exist, the deeper the sequencing depth, and the longer it takes. The number of variants is not only related to the sample itself, but also to the sequencing quality. The higher the quality, the faster the speed is. Figure 5B shows the relationship between the running time of DeteX and the average sequencing depth. It can be seen that there is a high linear relationship between them, and the correlation coefficient reached .8.

## Discussion

The performance of the Bayesian classification model depends on the probability of the variables, although this distribution model is usually used to detect all kinds of variations, and the actual NGS data is not ideally distributed. Furthermore, when there are many attributes or the attributes are highly correlated, the Bayesian classifier is also effective. This is because in NGS data, with the increase of subclone types, the statistical method of determining mutation based on the likelihood ratio tests becomes unstable. The sequencing errors and true variants are not absolutely distinguished. The reads and base anomalies in non-reference sequences may contain causes of both sequencing background noise and true variants. The Bayesian model is used in mutect, so that false-positive variations exists in the control samples. By filtering the control variation, the real tumor variation is deleted, and its sensitivity is reduced. The Bayesian model used in MuTect could filter out possible variants due to the low requirement resulting in non-variant loci in normal samples being considered as variant loci. MuTect can detect low-frequency variation, and its precision is high enough, but its sensitivity is not enough. Software that detects variants using Fisher’s exact test, such as VarDict and VarScan, is sufficient in sensitivity but not enough in precision. If both software are used to detect variants together, the number of false positive variants increases, which is a great challenge for clinical testing cycle time and accuracy. First, we used BC model to ensure the correct detection of high-frequency variation, then used FET algorithm to ensure the detection of low-frequency variation, and finally reduced false positives by various filtering conditions, thus ensuring high sensitivity and specificity of the test results.

When detecting substitution and complex variants, DeteX fully considers whether multiple adjacent variants occur simultaneously, which is expressed in the data as to whether they occur in the same reads. If two adjacent single nucleotide mutations occur simultaneously, they are considered as a substitution mutation which is written in the form of one variation, such as variation one in Table 3. If two mutations do not occur simultaneously, they are considered as two variants which are written in the form of two variants such as variation two or four in Table 3. Thus, this ensures the correctness of detection of substitution mutation. For complex mutations, the situation is similar. The adjacent gap, ins and SNV in the same reads are fully considered. They are combined into one variation, which ensures the accuracy of this mutation detection. Due to lack of local realignment and over-reliance on the comprision results, some of the variants that occur at the end of reads may be softclip off instead of being accurately compared, so the frequency of variant may be slightly lower, which needs to be improved.

DeteX method for detecting single sample mutations is the same as that of detecting somatic mutations in paired samples, except that there is no normal sample as a control. Therefore, it can detect low-frequency variation very well. Compared with the HaplotypeCaller in Gatk, which is commonly used to detect single sample mutations, DeteX is much more sensitive. This greatly improves the efficiency of detecting somatic mutations in tumor samples without control samples. It offers the possibility of reducing costs and provide testing for more patients.

## Conclusion

Compared to other softwares, DeteX has higher sensitivity and precision in detecting systemic SNV and InDel mutations in tumor samples, especially in the detecting substitution and complex variants. This enhances our confidence in detecting sparse driver mutations in tumor samples, reduces the workload of relevant staff and improves detection efficiency. Clinical applicable mutation detection software with high sensitivity and specificity is very important for patient therapy and clinical research. The advent of our software has led to significant advances in clinical genetic testing.

## Data Availability

The datasets presented in this study can be found in online repositories. The names of the repository/repositories and accession number(s) can be found in the article/Supplementary Material.

## Abbreviations

NGS, next-generation sequencing; SNVs, single nucleotide variants; MNVs, multi-nucleotide variants; InDels, insertion and deletions; FET, Fisher’s Exact Test; BC, Bayesian Classifier; VAFs, variant allele frequencies; PCR, polymerase chain reaction; BED, browser extensible data; AD, allele depth; AF, allele frequency; cfDNA, cell-free DNA.
